# Societal preferences for adjuvant melanoma health states: UK and Australia

**DOI:** 10.1186/s12885-017-3673-y

**Published:** 2017-10-17

**Authors:** Mark R. Middleton, Michael B. Atkins, Kaitlan Amos, Peter Feng Wang, Srividya Kotapati, Javier Sabater, Kathleen Beusterien

**Affiliations:** 1 University of Oxford Department of Oncology, Roosevelt Drive, Oxford, OX3 7DQ UK; 2 Georgetown-Lombardi Comprehensive Cancer Ctr, 3970 Reservoir Road, NW Research Building, Room E501, Washington, DC 20057 USA; 3 Bristol-Myers Squibb Co, 3401 Princeton Pike, Lawrenceville, NJ 08648 USA; 4 Bristol-Myers Squibb S.A. Quintanavides, 15, 28050 Madrid, Spain; 5 Outcomes Research Strategies in Health, Washington, DC 20008 USA

## Abstract

**Background:**

No studies have measured preference-based utility weights for specific toxicities and outcomes associated with approved and investigational adjuvant treatments for patients with resected high-risk melanoma.

**Methods:**

A cross-sectional study was conducted in the United Kingdom and Australia to obtain utilities for 14 adjuvant melanoma health states. One-on-one interviews were conducted using standard gamble; utility weights range from 0.0, dead, to 1.0, full health. Supplemental risk questions also were asked.

**Results:**

Among 155 participants (52% male; mean age, 46 years) “adjuvant treatment no toxicities” (0.89) was most preferred, followed by “induction treatment” (0.88), and “no treatment” (0.86). Participants least preferred “cancer recurrence” (0.62); the utility for “cancer recurrence and 10-year survival with treatment” was 0.70. Disutilities for grade 2 toxicities ranged from −0.06 for fatigue to −0.13 for hypophysitis. The mean maximum acceptable risk of a life-threatening event ranged from 30% for a 6% increase in the chance of remaining cancer free over 3 years to 40% for an 18% increase; Australian respondents were willing to take higher risks.

**Conclusion:**

Reproducible health utilities for adjuvant melanoma health states were obtained from the general population in two countries. These utilities can be incorporated into treatment-specific cost-effectiveness evaluations.

**Electronic supplementary material:**

The online version of this article (10.1186/s12885-017-3673-y) contains supplementary material, which is available to authorized users.

## Background

Currently, 132,000 melanoma skin cancers occur globally each year [1]. Malignant melanoma is the ninth most common cancer in Europe; in the United Kingdom, incidence rates are estimated to be the ninth highest among males in Europe and seventh highest among females [2]. In Australia, the incidence is the highest in the world due to the combination of high ultraviolet radiation, outdoor lifestyle, and a predominately Caucasian population [3]. In earlier-stage melanoma, the treatment of choice is surgical, and adjuvant therapy may be considered in patients with intermediate-risk melanoma [4]. Adjuvant melanoma therapies include high-dose interferon (IFN) α2b and low-dose pegylated-IFN [5]. More recently the anti-CTLA-4 antibody, ipilimumab, has been investigated in phase 3 trials [6] and received US FDA approval for treatment of patients with resected stage III melanoma in October 2015.

To evaluate the cost-effectiveness of therapies, particularly for life-threatening conditions such as cancer, it is useful to be able to assign cardinal utilities, or preference weights, to potential health outcomes in order to calculate quality-adjusted life expectancy. Health states that lend themselves to assignment of utility weights are those that impact patient health-related quality of life. In high-risk melanoma patients being considered for adjuvant therapy, such health states include treatment experience, toxicities, and relapse [7]. Consistent with the National Institute for Health and Care Excellence recommendations, preferences should be derived using a choice-based method, such as time trade-off or standard gamble, which typically value health states relative to full health and death. In addition, utilities ideally should be based on the general population perspective [8].

The standard gamble approach has been implemented in numerous studies in cancer [9–11]. Using this approach, a recent study focusing on health states associated with adjuvant IFN found that utilities for melanoma recurrence were significantly lower than for all IFN toxicity scenarios [9]. Another study used the standard gamble approach to identify utility values for 18 prostate cancer health states from the perspective of the general population as well as patients with prostate cancer [10]. Preference studies in cancer also have examined factors influencing treatment decision-making. A study using standard gamble to assess outcomes in metastatic colorectal cancer found that patients who were older, stage III versus IV and who had prior radiotherapy, lower educational attainment, and lower household income were less willing to tolerate treatment-related adverse events [11]. The humanistic impact, efficacy, and safety of treatment often are highlighted as the most important factors influencing treatment decision making among patients with cancer [12, 13].

Utility measurement in adjuvant melanoma to date has primarily focused on outcomes associated with IFN therapy [7, 14]. None have focused on adjuvant ipilimumab outcomes. As such, the objective of this study was to obtain utility weights for key outcomes, including toxicities and relapse, associated with ipilimumab and IFN in the adjuvant treatment of patients with high-risk melanoma.

## Methods

This was a cross-sectional study conducted in the United Kingdom and Australia to obtain utilities for adjuvant melanoma health states among adult members of the general public. Study participants were recruited through advertising by a market research company. One-on-one interviews were conducted with participants by trained interviewers using the standard gamble technique. In standard gamble, the respondent identifies the maximum risk of being dead that he or she is willing to take to avoid being in a selected health state. Specifically, respondents imagine that they are in a specific health state and can remain in that state or take a gamble that involves a chance (*p*) of achieving full health with a corresponding chance (1 − *p*) of being dead. The *p* probabilities are varied using a ping-pong approach, converging on *p* = 50%, until the respondent is indifferent to the two options [15]. A prop was used in each interview to help visualize the percentage risks. Study recruitment occurred from April through June, 2015. All participants provided informed consent and received compensation for their time. This study was approved by Magil Institutional Review Board (Rockville, MD) and complied with the tenets of the Declaration of Helsinki (Additional file 1).

Utilities were obtained for health states that included current health, five treatment-related states that included adjuvant treatment with no toxicity, induction treatment, no treatment, cancer recurrence, and recurrence with long term survival. The ‘long term survival’ state was developed based on findings showing a survival benefit associated with ipilimumab over 10 years [16]. Nine treatment toxicity states were described in association with receiving adjuvant cancer treatment. The toxicity states included key toxicities associated with ipilimumab (diarrhea, skin reaction/rash, hypophysitis), IFN (flu-like syndrome with myalgia/arthralgia, fatigue, depression), and nausea. The respective descriptions were developed based on the definitions of grade two events in the National Cancer Institute Common Toxicity Criteria for Cancer v4.0 [17]. To capture severe toxicities of any grade that result in an outpatient visit or hospitalization, two additional health states were developed to reflect these outcomes. To establish the context of the adjuvant treatment setting, all of the health states except for those describing cancer recurrence begin with: “You have undergone surgery and have had cancer completely removed, but you still are at high risk of the life-threatening cancer coming back.” “Melanoma” was not specified to minimize biased responses based on perceptions of this cancer.

All health states were labeled with symbols to avoid imposing a predetermined hierarchical order on the states. The descriptions were developed in layperson terms, and health states were refined with input from two clinical experts and a pilot test with 10 individuals from the general public (five in the United Kingdom and five in Australia). After the standard gamble exercise, the participants were asked three open-ended questions about the maximum acceptable risk they were willing to accept for a treatment with different levels of effectiveness. Specifically, they were asked: “If you had a life-threatening cancer, what is the maximum risk of a life-threatening event that you would be willing to accept to take a treatment that would increase your chance of remaining cancer free over 3 years by *X%*?” The three questions included 6%, 12%, and 18% as the effectiveness percentage. Finally, the respondents completed a form with questions on demographics and perceptions about health.

### Analysis

The target sample size for this study was to recruit approximately 85 individuals from each country in an effort to have analyzable data for 75 in each country. It was determined that 75 participants would be sufficient to yield estimates with standard errors as low as 0.03 [18]. All data were reported using descriptive statistics including means and frequencies, as applicable. For each health state, the respective utility equaled the probability *p* of full health at the point the respondent was indifferent to remaining in the health state and taking the gamble. Utility scores ranged from 0.0, reflecting being dead, to 1.0, reflecting full health. Disutilities for each of the toxicities were calculated by subtracting the utility for “adjuvant treatment, no toxicity” from the utility of the toxicity state. Statistical comparisons among subgroups were performed using analysis of variance and Pearson chi-square tests, as applicable; Tukey’s multiple comparison test was applied for comparisons across more than two subgroups. Statistical significance was determined based on a *p*-value of less than 0.05. SPSS (Version 22) was used to conduct all statistical analyses.

## Results

A total of 172 individuals participated in this research, 87 from the United Kingdom and 85 from Australia. Of the 172 respondents, 17 (9.8%) were excluded because they had at least three inconsistent pairs of standard gamble utilities; these included a) the utility for adjuvant treatment plus toxicity or severe toxicity was higher than the utility for adjuvant treatment without toxicity or b) the utility for cancer recurrence was higher than the utility of adjuvant treatment without toxicity. Excluded participants’ demographics did not differ from those of included participants, except for gender, in which females were excluded more than males (77% vs. 48%; *p* = 0.036). The total effective sample included 155 participants, 80 from the United Kingdom and 75 from Australia, residing in 43 cities across these countries.

Table 1 shows demographic and clinical characteristics for the study participants. The mean age was 46 ± 16 years, and 52% were male. The country-specific samples were closely matched according to the age and gender of the target adult populations in the United Kingdom and Australia, as reported in 2011 Census data [19, 20]. Although most respondents were Caucasian in both countries, more UK respondents were black (10% vs. 1%), and more Australians were Asian (13% vs. 2%). More Australians attained a higher level of education with a university or postgraduate degree (81% vs. 50%; *p* < 0.001) and were working full or part time (76% vs. 50%; *p* = 0.005). In response to a question inquiring about overall health, more Australian respondents reported being in “excellent” or “very good” health versus UK respondents (71% vs. 48%; *p* = 0.016). Similar percentages of participants reported having no health conditions. More Australian participants reported knowing someone with melanoma versus UK participants (29% vs. 7%; *p* < 0.001).

**Table 1:** Demographic characteristics

Characteristic	Overall (*N* = 155)	UK (*n* = 80)	Australia (*n* = 75)	*p* value
Age (±SD)	45.56 (±16.2)	46.1 (±17.8)	44.97 (±14.4)	0.66
Male	81 (52.3%)	40 (50%)	41 (54.7%)	0.56
Race				0.008
White	120 (77.4%)	65 (81.3%)	55 (73.3%)	
Black	9 (5.8%)	8 (10%)	1 (1.3%)	
Indian	8 (5.2%)	3 (3.8%)	5 (6.7%)	
Asian	12 (7.7%)	2 (2.5%)	10 (13.3%)	
Other/ multiracial	5 (3.2%)	2 (2.5%)	4 (5.3%)	
Employment				0.005
Full time	77 (49.7%)	34 (42.5%)	43 (57.3%)	
Part time	21 (13.5%)	7 (8.8%)	14 (18.7%)	
Retired Student	22 (14.2%) 14 (9%)	15 (18.8%) 11 (13.8%)	7 (9.3%) 3 (4.0%)	
Other	21 (13.5%)	13 (16.4%)	13 (10.6%)	
Education^a^				<0.001
Primary/junior/year 6	1 (0.6%)	1 (1.3%)	0	
Primary/third form/year 9	1 (0.6%)	0	1 (1.3%)	
Secondary/sixth form/year 13	51 (32.9%)	39 (48.8%)	12 (16%)	
University/college	80 (51.6%)	33 (41.3%)	47 (62.7%)	
Postgraduate degree	21 (13.5%)	7 (8.8%)	14 (18.7%)	
Overall health				0.016
Excellent	31 (20%)	12 (15.0%)	19 (25.3%)	
Very good	60 (38.7%)	26 (32.5%)	34 (45.3%)	
Good	39 (25.2%)	22 (27.5%)	17 (22.7%)	
Fair	17 (11%)	14 (17.5%)	3 (4.0%)	
Poor	8 (5.2%)	6 (7.5%)	2 (2.7%)	
Health conditions				
None	65 (41.9%)	30 (37.5%)	35 (46.7%)	0.248^b^
Arthritis	24 (15.5%)	16 (20%)	8 (10.7%)	
Heart disease	5 (3.2%)	5 (6.3%)	0	
Depression	22 (14.2%)	15 (18.8%)	7 (9.3%)	
Diabetes	6 (3.9%)	4 (5%)	2 (2.7%)	
GI problems	13 (8.4%)	6 (7.5%)	7 (9.3%)	
Pain	25 (16.1%)	15 (18.8%)	10 (13.3%)	
Respiratory disorder	23 (14.8%)	18 (22.5%)	5 (6.7%)	
Other	21 (13.5%)	8 (10%)	13 (17.3%)	
Experience with melanoma^a^				<0.001^c^
Been diagnosed with melanoma	7 (4.5%)	4 (5.0%)	3 (4.0%)	
Know someone with melanoma	28 (18.1%)	6 (7.5%)	22 (29.3%)	

^a^ Missing data for 1 respondent in Australia

^b^ *p*-value for difference in None category

^c^ *p*-value for difference for category “know someone with melanoma”

In response to the items about health perceptions, most participants (77%) reported that they would “rather live a short time in good health than a long time in very bad health.” However, most (86%) indicated that “if they had a life-threatening disease, they would do whatever to improve the chance of surviving,” and most (86%) would “accept feeling lousy for a year if it meant having a better chance of living longer.” Most (81%) reported having someone to take care of them. More Australian versus UK participants agreed that “it would be better to have cancer return after taking a treatment with strong side effects than to have it return without taking treatment” (40% vs. 31%; *p* = 0.007). More UK participants reported feeling downhearted and blue at least a little of the time during the past month (67% vs. 53%; *p* = 0.003).

### Preference weights

**Fig. 1:**
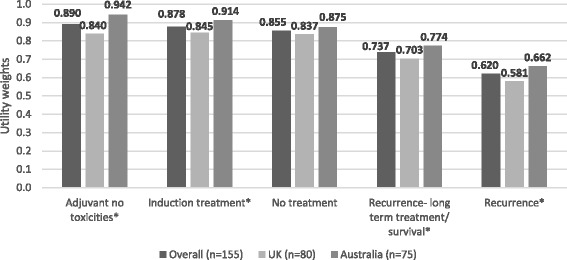
Mean standard gamble utilities for treatment-related health states. *p < 0.05 for differences between countries

**Fig. 2:**
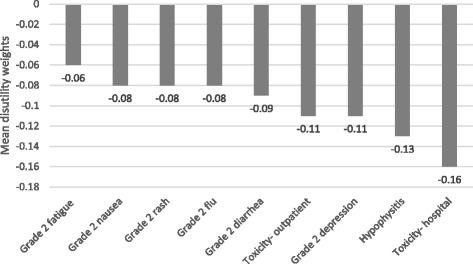
Mean disutilities for toxicity states: overall population

In Australia and the United Kingdom, utilities for “current health” were 0.99 and 0.97, respectively. Figure 1 shows the mean standard gamble utilities for the treatment-related states. “Adjuvant treatment no toxicities” (0.890) had the highest preference weight, followed by “induction treatment” (0.878), and “no treatment” (0.855). Whereas the Australian participants favored “adjuvant treatment no toxicities” (0.942) more than “no treatment” (0.875), the UK participants rated these states about the same (0.840 and 0.837 respectively). Participants in both the United Kingdom and Australia least preferred “cancer recurrence” (0.581 and 0.662, respectively) among all of the health states. The state describing cancer recurrence, but having a 10-year survival with treatment (“long-term survival”) had higher utilities than “cancer recurrence”; specifically, the utilities for “long-term survival” were 0.703 in the United Kingdom and 0.774 in Australia. Except for “no treatment,” the treatment-related state utilities were significantly higher (*p* < 0.05) among Australian respondents compared to UK respondents. The toxicity disutilities, including grade 2 toxicities and severe toxicities leading to an outpatient visit or hospitalization, are presented in Fig. 2; none differed significantly by country.

None of the standard gamble utility scores varied significantly by gender or race. Variations by age were not observed with the exception of the “no treatment” state, which was preferred more among those in the 18–39 age group compared to the 40–59 age group (0.914 vs 0.804; *p* = 0.005) (the mean utility for “no treatment” in the 60+ age group was 0.844). A comparison of utilities between those reporting “excellent” or “very good” health versus those reporting “good,” “fair,” or “poor” health in response to the question on overall health showed the former group to have a higher mean “current health” utility than the latter group (0.994 vs 0.959; *p* = 0.017). Otherwise, no other health state utilities differed significantly between these two groups. Individuals who completed college/university or above had significantly higher utilities compared to those with secondary/sixth form/year 13 or lower levels for “adjuvant treatment no toxicity,” “fatigue,” “diarrhea,” and “toxicity-outpatient.” Also, respondents knowing someone with melanoma had higher utilities for all of the health states versus those who did not know someone with melanoma (Table 2). The differences were significant for “adjuvant treatment no toxicities,” “induction treatment no toxicities,” “fatigue,” “rash,” “toxicity-outpatient,” and “long term survival.”

**Table 2:** Standard gamble scores by response to knowing someone with melanoma

	Know someone with melanoma (*n* = 28)	Do not know someone with melanoma (*n* = 122)	*p* value
Current health	0.989 ± 0.04	0.978 ± 0.08	0.507
Adjuvant treatment no toxicities	0.952 ± 0.09	0.877 ± 0.16	0.001
Induction treatment	0.941 ± 0.08	0.865 ± 0.17	0.001
No treatment	0.895 ± 0.16	0.846 ± 0.20	0.222
Fatigue	0.896 ± 0.13	0.821 ± 0.18	0.019
Nausea	0.873 ± 0.15	0.800 ± 0.20	0.078
Rash	0.885 ± 0.16	0.800 ± 0.20	0.040
Flu	0.853 ± 0.17	0.805 ± 0.18	0.195
Diarrhea	0.851 ± 0.19	0.785 ± 0.21	0.127
Toxicity-outpatient	0.886 ± 0.14	0.764 ± 0.21	0.001
Depression	0.831 ± 0.22	0.768 ± 0.23	0.154
Hypophysitis	0.818 ± 0.17	0.742 ± 0.21	0.078
Long-term survival	0.843 ± 0.19	0.715 ± 0.22	0.003
Toxicity-hospital	0.791 ± 0.18	0.726 ± 0.21	0.133
Recurrence of cancer	0.673 ± 0.26	0.607 ± 0.25	0.215

### Maximum acceptable risk

**Fig. 3:**
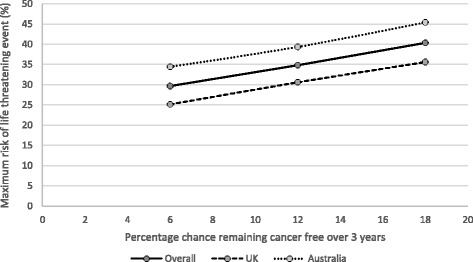
Risk-benefit tradeoff curve for UK, Australia, and overall

Figure 3 shows the mean maximum acceptable risk for each of the efficacy estimates by participants overall and by country. Overall, the mean maximum acceptable risk of a life-threatening event ranged from 30% for a 6% increase in the chance of remaining cancer free over 3 years, to 40% for an 18% increase. Australian respondents were more willing to take higher risks than UK respondents by approximately 9%; the difference was significant for an 18% increase in the chance of remaining cancer free (45.4% ± 29.0 vs. 35.6% ± 29.5; *p* = 0.039).

## Discussion

This study yielded general population utilities for adjuvant melanoma treatment-related health states, including those associated with IFN and ipilimumab. Utilities are reported for the United Kingdom, Australia, and the overall study population. With respect to the toxicities evaluated, disutilities (negative values) were calculated, facilitating the application of these values to patients who may be experiencing these effects, but may not currently be receiving adjuvant treatment. The utilities from this study can be used to quality-adjust life expectancy, as well as inform the evaluation of melanoma treatment outcomes in future studies.

The utilities obtained in this study are similar to those obtained by Kilbridge et al. [7], who used standard gamble to obtain utility weights for adjuvant IFN health states from patients with low-risk melanoma. Specifically, they obtained a mean utility for “IFN treatment without side effects” of 0.92; in this study, “adjuvant treatment without toxicity” had a mean utility of 0.89. The mean utilities for “cancer recurrence” in the Kilbridge et al. study and in this study were 0.61 and 0.62, respectively. Finally, “severe side effects” (that did not include hospitalization) had a mean utility of 0.81 in the Kilbridge et al. study; in this study, severe toxicity with an outpatient visit had a mean utility of 0.78.

As may be expected, the utilities obtained for “adjuvant treatment-no toxicities” and “recurrence” were higher in this study relative to comparable states in advanced melanoma reported by Beusterien et al. [21], which used standard gamble to obtain utilities for advanced melanoma treatment health states from the general UK and Australian populations. Specifically, the utilities for these states were approximately 0.10 points higher than those for “treatment of advanced melanoma with stable clinical response” and “recurrence of advanced melanoma,” respectively.

The disutilities for the toxicities, calculated by subtracting “adjuvant treatment no toxicity” from “adjuvant treatment plus toxicity,” were similar to the disutilities found for the same toxicity states in the Beusterien et al. [15] study. Specifically, the disutilities for the respective grade 2 toxicities in this study ranged from −0.08 (“rash,” “nausea,” and “flu-like syndrome”) to −0.09 (“diarrhea”). In the Beusterien et al. study, disutilities ranged from −0.06, for “skin reaction/rash” to −0.11 for “flu-like syndrome.” Also, in this study, the mean disutilities for “severe toxicity-outpatient” and “severe toxicity-hospital” were −0.11 and −0.16, respectively; in the Beusterien et al. study, the mean disutilities for these health states were −0.13 and −0.17, respectively. Among the toxicities in this study, hypophysitis was rated as worst. This may have been attributable to the verbiage in the health state description, which could be characterized as more severe than grade 2, as it referred to having headaches, blurred vision, and feeling sluggish.

Compared to the UK participants, the Australian participants reported higher preferences for all of the health states than the UK participants, except for “no treatment.” The higher preferences observed among the Australian participants may be in part attributable to higher familiarity with melanoma. Specifically, knowing someone with melanoma, which more often occurred among Australian participants, was associated with higher preferences for the health states. These participants may have higher preference for adjuvant treatment as well as adjuvant treatment coupled with toxicities because they have greater knowledge of the potentially devastating impact of life-threatening cancer and are thus willing to tolerate higher risks of side effects. This finding also is consistent with previous research that found that patients and caregivers tended to assign higher utilities for health states relative to the general population and non-caregivers, respectively [10, 22]. Age, gender, and perception of overall health largely were not associated with health state preferences, except that, as may be expected, those reporting “excellent” or “very good” health had more favorable utilities for the “current health” state versus those in “good,” “fair,” or “poor” health.

As expected, when asked to identify the maximum risk of a life-threatening side effect that was acceptable for a treatment that would increase the chance of survival over 3 years by 6%, 12%, or 18%, the study participants reported higher risk acceptance as treatment effectiveness increased. In agreement with our findings, more than 50% of low-risk melanoma patients found mild-moderate and severe IFN toxicity tolerable if accompanied by 4% and 10% improvements in 5-year survival [7].

As this study used a convenience sample of volunteers, it is unknown whether or not utilities among non-volunteers may differ. Also, it is unknown whether or not the magnitude of utility decrements for the toxicities was influenced by coupling the toxicities with taking adjuvant treatment. Nevertheless, this coupling more truly reflects reality. In addition, while our study did not consider health states with multiple toxicities, several studies have explored the estimation of utilities given this scenario and recommend using a minimal model in which joint-state utilities are predicted to be equivalent to the utility of the worse health state [23, 24]. If one was to use this model to interpret the current study findings, a patient receiving adjuvant treatment (average utility = 0.89) experiencing both grade 2 fatigue (average disutility = −0.06) and grade 2 depression (average utility = −0.11) would be assigned a utility of 0.78 (0.89 minus 0.11). The disutility of depression was incorporated instead of fatigue because the former has a lower utility.

## Conclusions

In summary, the findings from this study are consistent with previous preference research in melanoma, and the utilities obtained in this study may be used to quality-adjust life expectancy in adjuvant melanoma research. Because the methodology in this study is consistent with previous research in advanced melanoma, the utilities from these studies have the potential to be used together in research studies focusing on melanoma treatment across disease stages.

## Additional file

Additional file 1: Interview Script. (PDF 259 kb)

## Abbreviations

CTLA-4: cytotoxic T-lymphocyte-associated protein 4
FDA: Food and Drug Administration
GI: gastrointestinal
IFN: interferon
SD: standard deviation
UK: United Kingdom
US: United States
