# Supplementary material for: Societal preferences for adjuvant melanoma health states: UK and Australia
Source: BMC Cancer. 2017 Oct 17;17:689. doi: 10.1186/s12885-017-3673-y (PMC5646133; doi:10.1186/s12885-017-3673-y)
Supplement: Additional file 1: — Interview Script. (PDF 259 kb) [file 12885_2017_3673_MOESM1_ESM.pdf]

INTERVIEW SCRIPT

Standard Gamble Interview Script

Please note that the → below indicates when the Interviewer should perform a task.

On behalf of the research team, I would like to thank you for agreeing to participate in this study. To begin, I will spend a few minutes explaining the purpose of this survey and why we feel it's important to have your assistance.

All the information that you provide will be strictly confidential. Your participation is voluntary, and if I should come to a question you would rather not answer, just let me know and we will skip it.

It is important that we obtain opinions from people such as yourself because you may have different perceptions and experiences than health professionals. The purpose of this interview is to obtain your opinions about various health states. There are no right or wrong answers, and everyone's opinions differ on these matters.

As a first step, please review each of the health states and order them from most to least favorable.

[Once respondent has finished ordering the health states]

For each health state, I will ask you to decide between two choices: Choice A and Choice B. Choice A means that you would remain in a selected health state, and Choice B involves taking a chance. The amount of risk will be changed until we identify the point where it is difficult to choose between taking the chance or choosing to stay in the selected health state. In order to make the task easier to understand, we will use a prop.

→ SHOW EXAMPLE SLIDE 1

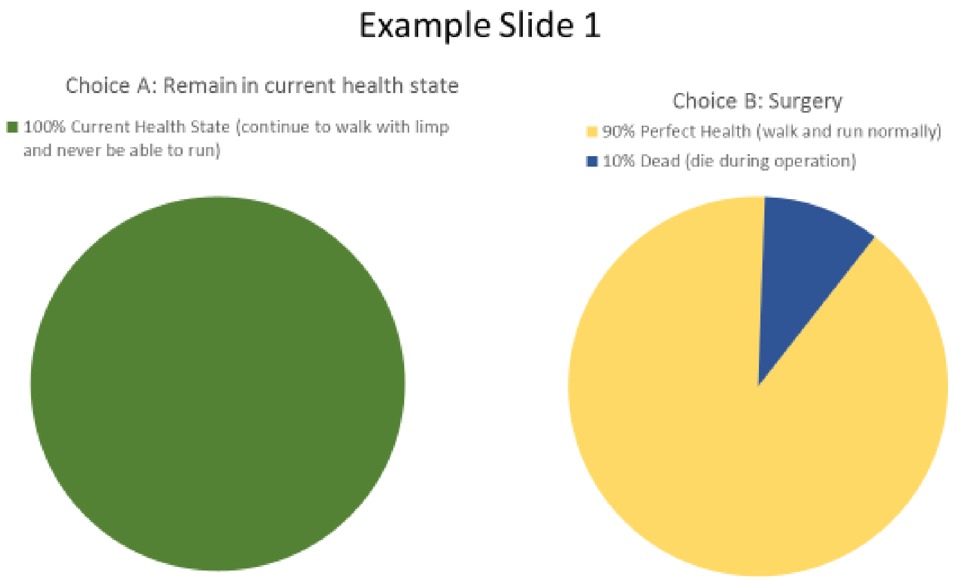

We call this a chance board because it indicates the chance or probability of an event occurring. As you can see, the left side of the board is labeled ‘Choice A’

→ POINT FINGER AT ‘CHOICE A’

The right part of the board is labeled ‘Choice B’

→ POINT FINGER AT ‘CHOICE B’

You will be asked to pick Choice A or Choice B. Choice A involves being in the selected health state, and this is a certainty. If something is certain it is equal to 100% chance or probability. Choice B involves taking a chance that has a certain probability of achieving perfect health with a corresponding probability of being dead. The chances of each of these results occurring are indicated by the size of the matching colors in the circle. Another way of explaining the chance aspect of Choice B is that for every 100 patients, 90 will become perfectly healthy with treatment.

→ POINT TO ‘90’

But 10 will die during treatment.

→ POINT TO ‘10’

And no one will know before choosing whether they will be one of the 90 or one of the 10. That is the chance they take. To make these ideas a little clearer lets run through a quick imaginary example. Let us assume you have been involved in an accident and you

hurt your leg. You now walk with a limp and you are unable to run. When you see a doctor, he/she explains that you have a choice of an operation.

→ SHOW EXAMPLE SLIDE 1

Choice A is to continue to walk with a limp and never be able to run. Choice B is an operation. Choice B in this example in this example, is risky. You could die during the operation. If you survive the operation, your leg is fixed and you can walk and run normally. The percent chance of surviving the operation and walking and running normally is shown above and is also represented by the proportion of yellow color in the circle.

Here, the chances of surviving the operation and running normally are 90% with a 10% chance of dying from the operation. If I were to spin an imaginary arrow, chances are it will probably land on yellow because there is a very large amount of yellow, 90 percent, showing in the circle. There is still a chance that it might land on blue, but it is a smaller chance, because there is only a small amount of blue showing on the circle. Another way to think of it is that, on average, for every 100 people who choose the operation, 90 will survive the operation and walk and run normally, and 10 will die from the operation.

→ POINT TO BLUE CHANCE VALUE AND BLUE SECTION OF THE CIRCLE

The chance of dying from the operation is shown in blue in the description and matches the proportion of blue color on this circle.

The chances will change and I will ask you to choose Choice A or Choice B each time. Or, please tell me if you think it is too difficult to choose between Choice A versus Choice B; in other words, the two choices are equal in your view.

→ SHOW EXAMPLE SLIDE 2; POINT TO CHOICE B

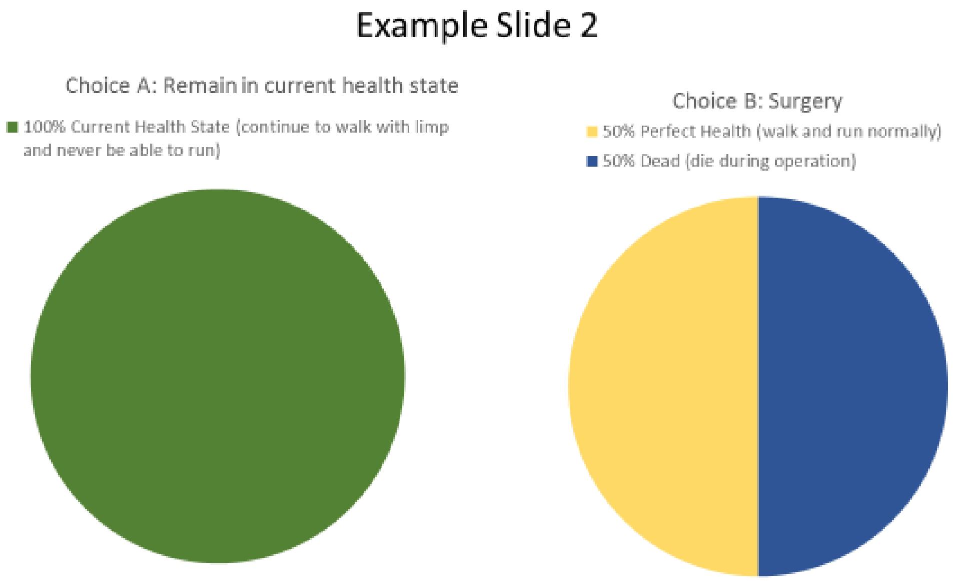

Now I've changed the chances. Now if I were to spin the imaginary arrow, what color is the arrow most likely to stop on?

→ ALLOW RESPONDENT TO ANSWER

There is an equal chance that it will stop on yellow or blue, because there is an equal amount of yellow and blue showing on the circle. Do you understand how the chance board works?

→ IF YES, CONTINUE BELOW. IF NO, RETURN TO THE BEGINNING OF THE CHANCE SECTION AND REPEAT THE EXERCISE.

Let's work through the first question carefully together. In Choice A, there is a 100% chance you will remain in your this health state shown in green. In Choice B the yellow shows chance Perfect Health, and the chance of being dead is shown in blue.

Begin Exercise

→ HAND RESPONDENT A HEALTH STATE DESCRIPTION CARD

Please read over the description…

→ BE SURE WHEEL (CHOICE B) IS 100/0

We are now ready to begin. Please imagine that you are in health state [insert symbol]. You may choose to stay in health state [insert symbol], or you can take a chance, which is Choice B. If you select choice B you have a 100% chance of perfect health and 0% chance of death. Which would you choose—Choice A or B?

Responses

→ A- Why did you choose 100 percent chance of this health state rather than 100 percent chance of perfect health? RECORD VERBATIM RESPONSE IN RESPONSE SHEET. CLARIFY IF RESPONDENT UNDERSTANDS EXERCISE; IF YES, GO TO NEXT HEALTH STATE; IF NO, REPEAT QUESTION.

B- GO TO WHEEL 0/100 AND CONTINUE BELOW

Now I've changed Choice B to show that there is a 0% chance of perfect health and 100% chance of death. Choice A is still a 100% chance of remaining in this health state. Choice B involves a gamble. Would you choose A or B?

→ A- GO TO WHEEL 95/5, CONTINUE BELOW

B- PROMPT: Do you mean you would prefer to have a 100% chance of being dead and a 0% chance of perfect health rather than living in this health state? RECORD ANSWER ON RESPONSE SHEET

NO- REPEAT CHOICES SHOWN ON BOARD

The board now shows that Choice B is 95% perfect health and 5% chance of being dead, and Choice A is to stay in this health state. Which would you choose—Choice A or B?

→ A- CONTINUE THE QUESTIONS DOWN TO A 1% INCREMENTAL CHANGE (respondent is not willing to take a 5% chance of being dead for 95% chance of perfect health— thus interviewer should ask is choice B were 96% chance of perfect health/ 4% chance being dead [no= record response, yes= continue 97%/3%, 98%/2%, 99%/1%]
GO TO NEXT QUESTION

B- GO TO WHEEL 5/95, CONTINUE BELOW

The board now shows Choice B to be a 5% chance of perfect health with a 95% of being dead, and Choice A is to stay in this health state. Which would you choose—Choice A or B?

→ A-GO TO WHEEL 90/10, CONTINUE BELOW

B- CONTINUE THE QUESTIONS DOWN TO A 1% INCREMENTAL CHANGE.
(respondent is willing to take a 95% chance of being dead and a 5% chance of perfect health; thus, interviewer should ask if Choice B were 4% chance of being dead/96% perfect health; 3%/97%; 2%/98%, and 1%/99%)
GO TO NEXT QUESTION

The board now shows Choice B to be a 90% chance of perfect health with a 10% chance of being dead, and Choice A is to stay in this health state. Which would you choose—Choice A or B?

→ A- CONTINUE THE QUESTIONS DOWN TO A 1% INCREMENTAL CHANGE.
(respondent is willing to take a 5% chance of being dead but not a 10% chance of being dead; thus, interviewer should ask if Choice B were 91% chance of perfect health/9% dead; 92%/8%; 93%/7%, and 94%/6%)
GO TO NEXT QUESTION

B- GO TO WHEEL 10/90, CONTINUE BELOW

The board now shows Choice B to be 10% of perfect health with a 90% chance of being dead, and Choice A is to stay in this health state. Which would you choose—Choice A or B?

→ A- GO TO WHEEL 85/15, CONTINUE BELOW

B- CONTINUE THE QUESTIONS DOWN TO A 1% INCREMENTAL CHANGE.
(respondent is not willing to take a 95% chance of being dead but is willing to take a 90% chance of being dead; thus, interviewer should ask if Choice B were 9% chance of perfect health/91% dead; 8%/92%; 7%/93%, and 6%/94%)
GO TO NEXT QUESTION

The board now shows Choice B to be an 85% chance of perfect health with a 15% change of being dead, and Choice A is to stay in this health state. Which would you choose—Choice A or B?

→ A- CONTINUE THE QUESTIONS DOWN TO A 1% INCREMENTAL CHANGE.
GO TO NEXT QUESTION

B- GO TO WHEEL 15/85, CONTINUE BELOW

The board now shows Choice B to be a 15% of perfect health with an 85% chance of being dead, and choice A is to stay in this health state. Which would you choose—Choice A or B?

→ A- GO TO WHEEL 80/20, CONTINUE BELOW
B- CONTINUE THE QUESTIONS DOWN TO A 1% INCREMENTAL CHANGE.
GO TO NEXT QUESTION

The board now shows Choice B to be an 80% chance of perfect health with a 20% chance of being dead, and Choice A is to stay in this health state. Which would you choose—Choice A or B?

→ A- CONTINUE THE QUESTIONS DOWN TO A 1% INCREMENTAL CHANGE
GO TO NEXT QUESTION

B- GO TO WHEEL 20/80, CONTINUE BELOW

The board now shows Choice B to be a 20% chance of perfect health with an 80% chance of being dead and Choice A is to stay in this health state. Which would you choose—Choice A or B?

→ A- GO TO WHEEL 75/25, CONTINUE BELOW

B- CONTINUE THE QUESTIONS DOWN TO A 1% INCREMENTAL CHANGE
GO TO NEXT QUESTION

The board now shows Choice B to be a 75% chance of perfect health with a 25% chance of being dead, and Choice A is to stay in this health state. Which would you choose—Choice A or B?

→ A- CONTINUE THE QUESTIONS DOWN TO A 1% INCREMENTAL CHANGE.
GO TO NEXT QUESTION

B- GO TO WHEEL 25/75, CONTINUE BELOW

The board now shows choice B to be a 25% chance of perfect health with a 75% chance of being dead, and Choice A is to stay in this health state. Which would you choose—Choice A or B?

→ A- GO TO WHEEL 70/30, CONTINUE BELOW

B- CONTINUE THE QUESTIONS DOWN TO A 1% INCREMENTAL CHANGE
GO TO NEXT QUESTION

The board now shows Choice B to be a 70% chance of perfect health with a 30% chance of being dead, and Choice A is to stay in this health state. Which would you choose—Choice A or B?

→ A- CONTINUE THE QUESTIONS DOWN TO A 1% INCREMENTAL CHANGE.
GO TO NEXT QUESTION

B- GO TO WHEEL 30/70, CONTINUE BELOW.

The board now shows Choice B to be a 30% chance of perfect health with a 70% chance of being dead, and Choice A is to stay in this health state. Which would you choose—Choice A or B?

→ A- GO TO WHEEL 65/35, CONTINUE BELOW

B- CONTINUE THE QUESTIONS DOWN TO A 1% INCREMENTAL CHANGE. GO TO NEXT QUESTION

The board now shows Choice B to be a 65% chance of perfect health with a 35% change of being dead, and Choice A is to stay in this health state. Which would you choose—Choice A or B

→ A- CONTINUE THE QUESTIONS DOWN TO A 1% INCREMENTAL CHANGE. GO TO NEXT QUESTION

B- GO TO WHEEL 35/65, CONTINUE BELOW

The board now shows Choice B to be a 35% chance of perfect health with a 65% chance of being dead, and Choice A is to stay in this health state. Which would you choose—Choice A or B?

→ A- GO TO WHEEL 60/40, CONTINUE BELOW

B- CONTINUE THE QUESTIONS DOWN TO A 1% INCREMENTAL CHANGE. GO TO NEXT QESTION

The board now shows Choice B to be a 60% chance of perfect health with a 40% chance of being dead, and Choice A is to stay in this health state. Which would you choose—Choice A or B?

→ A- CONTINUE THE QUESTIONS DOWN TO A 1% INCREMENTAL CHANGE. GO TO NEXT QUESTION

B- GO TO WHEEL 40/60, CONTINUE BELOW

The board now shows Choice B to be a 40% chance of perfect health with a 60% chance of being dead, and Choice A is to stay in this health state. Which would you choose—Choice A or B?

→ A- GO TO WHEEL 55/45, CONTINUE BELOW

B- CONTINUE THE QUESTIONS DOWN TO A 1% INCREMENTAL CHANGE. GO TO NEXT QUESTION

The board now shows Choice B to be a 55% chance of perfect health with a 45% chance of being dead, and Choice A is to stay in this health state. Which would you choose—Choice A or B

→ A-CONTINUE THE QUESTIONS DOWN TO A 1% INCREMENTAL CHANGE. GO TO NEXT QUESTION

B- GO TO WHEEL 45/55, CONTINUE BELOW

The board now shows Choice B to be a 45% change of perfect health with a 55% chance of being dead, and Choice A is to stay in this health state. Which would you choose—Choice A or B?

→ A- GO TO WHEEL TO 50/50, CONTINUE BELOW

B- CONTINUE THE QUESTIONS DOWN TO A 1% INCREMENTAL CHANGE. GO TO NEXT QUESTION

The board now shows Choice B to be a 50% chance of perfect health with a 50% chance of being dead, and Choice A is to stay in this health state. Which would you choose—Choice A or B

→ A- CONTINUE THE QUESTIONS DOWN TO A 1% INCREMENTAL CHANGE.
(respondent is willing to take a 45% chance of being dead but not a 50% chance of being dead; thus, interviewer should ask if Choice B were 54% chance of perfect health/46% dead; 53%/47%; 52%/48%, and 51%/49%)

B- MARK RESPONSE

→ EXERCISE IS REPEATED FOR EACH HEALTH STATE
